# Predicting Microbial Growth Dynamics in Commercial Cocoa‐Flavored Plant‐Based Milk Alternatives

**DOI:** 10.1111/1750-3841.71189

**Published:** 2026-06-09

**Authors:** Clara Mariana Gonçalves Lima, Jaqueline Sousa Correia, Dionísio Pedro Amorim‐Neto, Jolanta Wawrzyniak, Anderson S. Sant'Ana

**Affiliations:** ^1^ Department of Food Science and Nutrition, Faculty of Food Engineering University of Campinas Campinas Brazil; ^2^ Faculty of Food Science and Nutrition Poznań University of Life Sciences Poznań Poland

**Keywords:** bacterial growth model, growth potential, microbiological safety, new foods, pathogenic bacteria, plant‐based beverages

## Abstract

This study evaluated the growth of *Bacillus cereus*, *Escherichia coli*, and *Salmonella* Typhimurium in commercially available cocoa‐flavored plant‐based milk alternatives (PBMAs) under different temperatures and examined the influence of product composition on bacterial development. Bacterial populations were monitored over 12 h at 25°C for all strains and additionally at their optimal growth temperatures (30°C for *B. cereus*, 37°C for *E. coli*, and *S*. Typhimurium). The nutritional composition of the PBMAs was analyzed, and principal component analysis (PCA) was performed to identify key compositional factors. Results showed that the increase in *E. coli* and *S* *almonella* Typhimurium population was significantly higher at 37°C than at 25°C on all cocoa‐flavored PBMAs (*p* < 0.05), whereas for *B. cereus*, the differences between 25°C and 30°C were relatively small, with statistical significance observed only for Substrate A (*p* < 0.05). Growth was significantly influenced by bacterial species (MS = 10.49, *p* < 0.001), substrate composition (MS = 0.48, *p* < 0.001), and interaction of both these factors (MS = 0.29, *p* = 0.001). The formulated regression models based on environmental factor (temperature), PCA scores (reflecting substrate composition, particularly protein, fiber, fat, sodium, and energy balance, and for *B. cereus* also sodium, fiber, and pH‐related properties), and time, accurately reproduced bacterial growth (*R* ^2^ = 0.857–0.898), highlighting the combined effects of these factors. By mapping new PBMA formulations onto the tested compositional space, it may also support contamination risk assessment and contribute to ensuring consumer safety.

**Practical Applications:**

The findings highlight that cocoa‐flavored plant‐based milk alternatives can support the growth of microorganisms depending on their composition and storage temperature. Understanding how nutrients such as protein, fiber, fat, and sodium drive microbial proliferation enables manufacturers to optimize formulations for improved microbial safety. The regression models developed can support predictive assessments during product design. These insights can guide industry and regulators in establishing safer handling, storage, and formulation strategies for PBMAs.

## 1 Introduction

The consumption of plant‐based foods has increased markedly in recent decades, driven by environmental concerns, ethical considerations regarding animal welfare, and the rising prevalence of food intolerances and allergies, such as lactose intolerance and cow's milk protein allergy. Within this trend, plant‐based milks, or plant‐based milk alternatives (PBMAs), have emerged as a rapidly expanding segment of the market, offering alternatives to conventional cow's milk (Kasapidou et al. 2023; Lee et al. 2024; McClements et al. 2019; Vogelsang‐O'Dwyer et al. 2021). These beverages are typically produced through aqueous extraction of plant materials, followed by filtration, homogenization, and, in some cases, nutritional fortification and the addition of stabilizers and flavorings to improve their sensory and nutritional properties (Jeske et al. 2018; Sethi et al. 2016). The raw materials used in their production, ranging from cereals (rice, oats, and corn) and legumes (soy and pea) to nuts (almond, cashew, and coconut) and pseudocereals (quinoa and amaranth), confer diverse nutritional and sensory profiles, though protein content and bioavailability may vary significantly compared to cow's milk. Consequently, PBMAs derived from these sources may also offer additional health‐promoting compounds, such as fibers, polyphenols, and unsaturated fatty acids (Grau‐Fuentes et al. 2023; Irondi et al. 2025; Paul et al. 2020).

The global dairy alternatives market is experiencing rapid growth, being valued at $34.59 billion in 2025 and projected to increase from $38.63 billion in 2026 to approximately $102.74 billion by 2035, at a CAGR of 11.50% (Precedence Research 2025). Despite their popularity, the shelf life and microbial safety of PBMAs remain critical issues, requiring further investigation, especially in the context of climate‐driven temperature fluctuations that may increase non‐sterility events during distribution and storage (Misiou et al. 2023). In addition, deviations from recommended storage conditions, such as temperature abuse during transportation, retail display, or domestic handling, may create favorable environments for microbial growth, particularly in products with heterogeneous compositions. These factors highlight the need for a deeper understanding of how intrinsic (composition, pH, and water activity) and extrinsic (temperature and other storage conditions) variables interact to influence microbial behavior in PBMAs.

PBMAs can be contaminated at multiple points along the production chain, from the use of contaminated plant‐based raw materials to postharvest handling, processing, storage, and distribution (Mukuna et al. 2021). Previous studies have demonstrated the presence of diverse microbial populations in plant‐derived ingredients, highlighting their role as potential sources of contamination (Kyrylenko et al. 2023; Amorim‐Neto et al. 2025; Lima et al. 2025; Gonçalves Lima et al. 2026).

In this context, enteric microorganisms such as *Salmonella* spp. and *Escherichia coli* are of particular concern in plant‐based food systems due to their frequent association with raw plant materials, including cereals, legumes, and nuts. Contamination may occur at multiple stages, including cultivation, harvesting, processing, and handling, often linked to environmental exposure to soil, irrigation water, or animal sources. These microorganisms are capable of surviving under adverse conditions and may persist throughout processing, subsequently proliferating when environmental conditions become favorable. The production of toxins and virulence factors is responsible for the pathogenicity of these bactéria (Abebe et al. 2020). Their presence in plant‐based ingredients highlights the potential for contamination of PBMAs, particularly when processing or storage conditions are inadequate.

In addition to non‐spore‐forming pathogens, *Bacillus cereus* represents a significant concern in PBMAs due to its ability to form highly resistant endospores. Commonly found in soil and plant‐derived raw materials, B. cereus can survive thermal treatments, including UHT processing, allowing it to persist in the final product. Under favorable environmental conditions, these spores may germinate and lead to vegetative growth, potentially resulting in spoilage and the production of toxins associated with foodborne illness. Consequently, even UHT‐treated PBMAs may harbor low levels of pathogenic microorganisms capable of proliferating under temperature abuse or inadequate storage conditions (Kain et al. 2024).

The presence of cocoa and sugars alters water activity, pH, and nutrient availability, potentially influencing the survival and proliferation of pathogenic microorganisms in these beverages. Despite their growing diversity and complexity, there is still limited knowledge regarding the growth dynamics of foodborne pathogens in cocoa‐flavored formulations, particularly considering the combined effects of product composition and storage temperature. Addressing this gap is crucial for ensuring consumer safety and guiding processing and storage practices. Therefore, this study evaluated the growth of *Salmonella* Typhimurium, *E. coli*, and *B. cereus* in commercially available cocoa‐flavored PBMAs subjected to UHT treatment, both at their optimal growth temperatures (37°C for *S* *almonella* Typhimurium and *E. coli*; 30°C for *B. cereus*) and at room temperature (25°C). The results provide valuable insights into the behavior of microorganisms in this emerging food category.

## 2 Materials and Methods

### 2.1 Sample Acquisition

Commercial UHT cocoa‐flavored plant‐based beverages from five different brands (A–E), hereafter referred to as media (i.e., beverage samples), were purchased in Campinas, São Paulo, Brazil, and transported to the Quantitative Food Microbiology Laboratory at the State University of Campinas. Each brand corresponded to a distinct formulation, with the following base ingredients: medium A—cashew and cocoa; medium B—oat and cocoa; medium C—almond and cocoa; medium D—pea and cocoa; and medium E—cashew and cocoa. The products were stored at room temperature until further analysis. Prior to opening, the packages were disinfected with 70% ethanol. All label information was transcribed into an electronic spreadsheet.

### 2.2 Bacterial Strains and Culture Conditions

#### 2.2.1 Culture of Strains

*S* *almonella* Typhimurium (ATCC 14028), *B. cereus* (NCTS 11143), and *E. coli* (ATCC 11229) were individually cultured in tryptic soy broth (TSB) (Merck, Germany). Cultures were incubated for 24 h at 30°C for *B. cereus* and at 37°C for the other strains. Subsequently, each strain was streaked onto tryptic soy agar (TSA) (KASVI, Brazil) and incubated for 18–20 h.

#### 2.2.2 Inoculum Preparation

Colonies were suspended in 0.85% (w/v) sterile saline solution until reaching a turbidity equivalent to 0.5 on the McFarland scale (Correia et al. 2026; ANVISA 2005), corresponding to the target microbial concentration (∼1.5 × 10^8^ cfu/mL).

#### 2.2.3 Inoculation of Cocoa‐Flavored Plant‐Based Beverages

Aliquots (30 mL) of cocoa‐flavored plant‐based beverages were inoculated with 100 µL of the prepared bacterial suspension (v/v). The samples were incubated at 37°C, 30°C, or 25°C for up to 12 h, and microbiological analyses were performed at 0, 6, and 12 h. Prior to inoculation, the beverages were tested for background microbial contamination.

#### 2.2.4 Microbiological Quantification

Samples were serially diluted in 1% (w/v) peptone water (Sigma‐Aldrich, Brazil) and plated onto selective agar media for enumeration of the target pathogens. *S* *almonella* Typhimurium was quantified on xylose lysine deoxycholate (XLD) agar (Neogen, Brazil), *B. cereus* on Mannitol Egg Yolk Polymyxin (MYP) agar (Sigma‐Aldrich, Brazil), and *E. coli* on MacConkey agar (Merck, Germany). Plates were incubated at 30°C for *B. cereus* and at 37°C for the other pathogens for 24–48 h. Results were expressed as log_10_ (cfu/mL), with a method quantification limit of 2 log_10_ (cfu/mL).

### 2.3 Growth Potential in Cocoa‐Flavored Plant‐Based Beverages

The growth potential (*δ*) of the pathogens under study in cocoa‐flavored PBMAs was determined using Equation (1), calculated as the differences between microbial counts (log_10_ cfu/mL) at the end of the experiments (after 12 h of incubation) and at time zero (immediately following inoculation). The beverages were considered favorable substrates for microbial growth when *δ* exceeded 0.5 log_10_ (cfu/mL), which corresponds to the uncertainty threshold of microbiological methods (AFSSA 2004; EURL Lm 2008).

(1) $$\delta =\log N_{f}-\log N_{0}$$

with *N*_f_ and *N*_0_ representing the final and initial microbial counts (cfu/mL) during the storage period.

### 2.4 Physicochemical Analysis

The pH of the samples was determined by homogenizing 10 mL of each sample and measuring with a previously calibrated digital pH meter (Model K39–2014B, KASVI, China). All analyses were performed in triplicate, and results were expressed as mean values (IAL 2005).

### 2.5 Statistical Analysis

Statistical analyses were performed using Statistica 13.3 (TIBCO Software Inc., Palo Alto, CA, USA). The experimental data are presented as mean ± standard deviation (SD) from three independent experiments. To assess the effects of bacterial species and the brand of cocoa‐flavored PBMAs on the increase in bacterial population levels, a two‐factor analysis of variance (ANOVA) was conducted after 12 h of incubation at 25 T°C. For this purpose, a full factorial design was used with factors: bacterial species (3 levels) and PBMA brand (5 levels), resulting in 15 experimental combinations (3 × 5). Each combination was performed in three independent biological replicates. Prior to analysis, assumptions of normality and homogeneity of variance within subgroups were verified using the Shapiro–Wilk test and homogeneity of variances using Levene's test. The analysis evaluated the main effects of bacterial species, PBMA brand, and their interaction. Post hoc comparisons were performed using Tukey's test to identify homogeneous groups. A significance threshold of *p* = 0.05 was applied.

### 2.6 Principal Component Analysis (PCA)

The PCA analysis, on the basis of 10 input variables describing the physicochemical characteristics of the PBMA substrates (total fat (TotFat), total protein (TotProt), total fiber (TotFib), total carbohydrates (TotCarb), total sugars (TotSugars), added sugars (AddSugars), calcium, total sodium (TotSod, EV kcal, pH), was used to explore similarities and differences between the tested media. PCA was carried out using the dedicated module in the Statistica 13.3 (TIBCO Software Inc., Palo Alto, CA, USA) package. Prior to the analysis, all variables were standardized to ensure comparability by eliminating the effect of differences in measurement scales. For this purpose, *Z*‐score normalization was applied, involving the subtraction of the mean and division by the SD for each variable as follows:

(2) $$z_{i}=\frac{x_{i}-{\bar{x}}_{i}}{\mathrm{SD}}$$

where *x_i_* is the value of variable, ${\bar{x}}_{i}$ is the mean variable value, and SD is the standard deviation. Then for each variable, principal component scores were computed using the regression‐based method implemented in used software, based on factor score coefficients derived from the training dataset. Consequently, these coefficients define the linear transformation from standardized compositional variables to the principal component space. The number of principal components retained for variable interpretation was determined on the basis of the scree plot (eigenvalue distribution) and the cumulative percentage of total variance explained. All selected components that accounted for a substantial proportion of the variability in the dataset were located before the inflection point of the scree plot. The results were visualized using score plots and biplots, which allowed for a simultaneous presentation of PBMA samples distribution according to the selected principal components and the contribution of the original variables to the formation of these components. This approach facilitated the identification of groups of tested matrix with similar characteristics, as well as variables most responsible for the observed differentiation.

### 2.7 Regression Analysis

Multiple linear regression models were developed to estimate the proliferation of each investigated bacterial strain in relation to environmental and sample‐related factors. The general structure of these models describes bacterial population increase as a function of time, temperature, and samples composition, with the latter represented by the three principal components (Equation 2):

(3) $$\Delta \log N=\beta_{0}+\beta_{1}\cdot \;\tau +\beta_{2}\cdot T+\beta_{3}\cdot \mathrm{PC}1+\beta_{4}\cdot \mathrm{PC}2+\beta_{5}\cdot \mathrm{PC}3+\varepsilon$$

where *ΔLog* *N* is the increase in the logarithm of the bacterial load (log_10_ cfu/mL), *τ* is the time (h), *T* is the temperature (°C), *PC1*, *PC2*, *PC3* are principal components representing sample composition, *β*_0_ *, β*_1_,…,*β*_5_ are regression coefficients, and *ε* is random error term.

Regression coefficients were estimated for each bacterial strain, and their significance was evaluated using standard errors and *p* values. Model performance was assessed by plotting observed versus predicted values and calculating correlation coefficients (*R*) and coefficients of determination (*R* ^2^). Additionally, the accuracy of the model was evaluated using RMSE (root mean square error) and MAE (mean absolute error).

## 3 Results and Discussion

**FIGURE 1:**
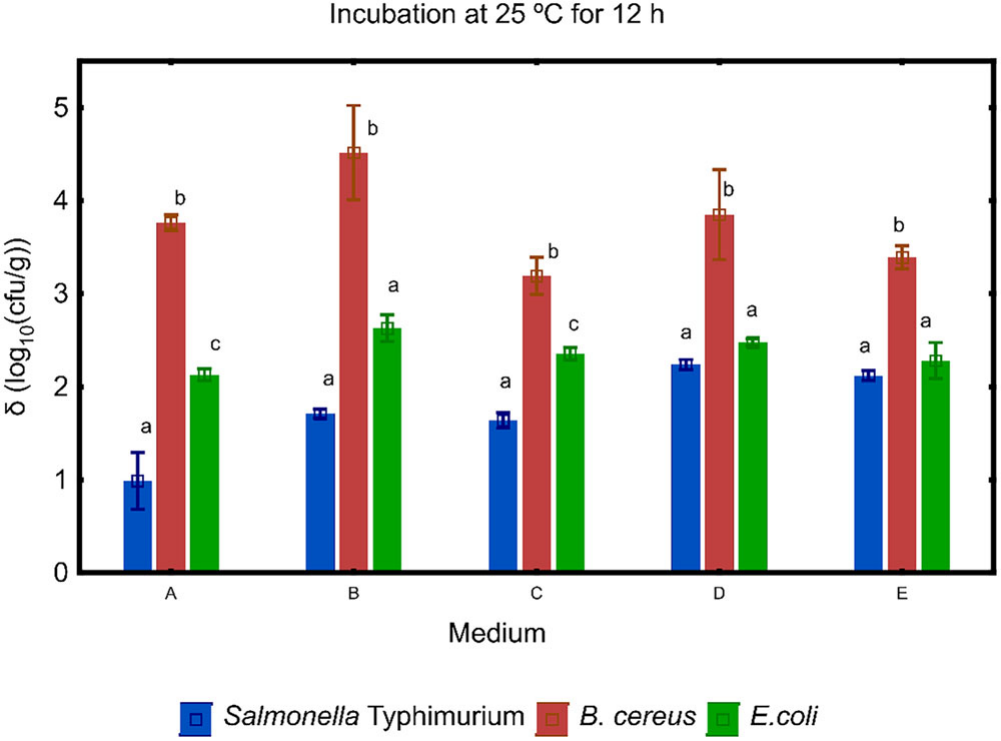
Comparison of the increase in the populations of bacteria (*S* *almonella* Typhimurium, *B*. *cereus*, and *E*. *coli*) during 12‐h incubation at 25°C on different cocoa‐flavored PBMAs. Distinct letter labels within each substrate denote statistically significant differences (*p* < 0.05) in the increase of bacterial levels across the examined strains.

A comparison of the growth dynamics of the tested bacterial species in the cocoa‐flavored plant‐based beverage at 25°C showed that B. cereus exhibited the most intensive expansion, whereas the strains of E. coli and *S* *almonella* Typhimurium showed significantly lower increases, with *S* *almonella* Typhimurium displaying the least pronounced population rise (Figure 1). The experimental results indicated that bacterial growth dynamics are likely associated with the strain type and its interactions with the composition of the matrix.

The more pronounced growth of *B. cereus* observed in this study can be attributed to its distinct physiological and metabolic characteristics. Although vegetative cells were used as inoculum, *B. cereus* is known for its ability to form spores, which contributes to its ecological persistence and rapid adaptation to favorable environments. In nutrient‐rich matrices such as PBMAs, the observed growth is primarily associated with its high metabolic versatility. *B. cereus* produces a wide range of extracellular enzymes, including proteases, amylases, and lipases, enabling efficient degradation and utilization of complex substrates present in plant‐based systems. This enzymatic capacity provides a competitive advantage over non‐spore‐forming bacteria *E. coli* and *S*almonella Typhimurium, particularly in nutrient‐dense substrates. Furthermore, *B. cereus* exhibits relatively broad tolerance to environmental factors such as pH and osmotic conditions, which may contribute to its ability to maintain active growth under varying physicochemical conditions of cocoa‐flavored PBMAs.

The interpretation regarding interactions of strain with the composition of the matrix is consistent with previous studies reporting variability in occurrence and growth behavior of *B. cereus* across different substrates. *B. cereus* contamination has been reported in raw materials and certain base products used for plant‐based beverages, such as oat flour and pea or oat syrups (Gleissle et al. 2025). In contrast, Butovskaya et al. (2025) found no evidence of *B. cereus* contamination in oat, almond, or rice beverages. In another study, members of the *B. cereus* group were isolated from soy‐ and oat‐based PBMAs, with counts of 1 and 2 cfu/mL, respectively (Kain et al. 2024). These findings support the hypothesis that PBMAs can be contaminated by this microorganism and highlight the ability of *B. cereus* strains to persist across a wide temperature range. In line with this, Champidou et al. (2024) showed that *Bacillus* *licheniformis* and *Bacillus* *subtilis* spores display greater heat resistance in pea‐based milk alternatives compared to dairy milk, underscoring the importance of the *Bacillus* group in such products.

**FIGURE 2:**
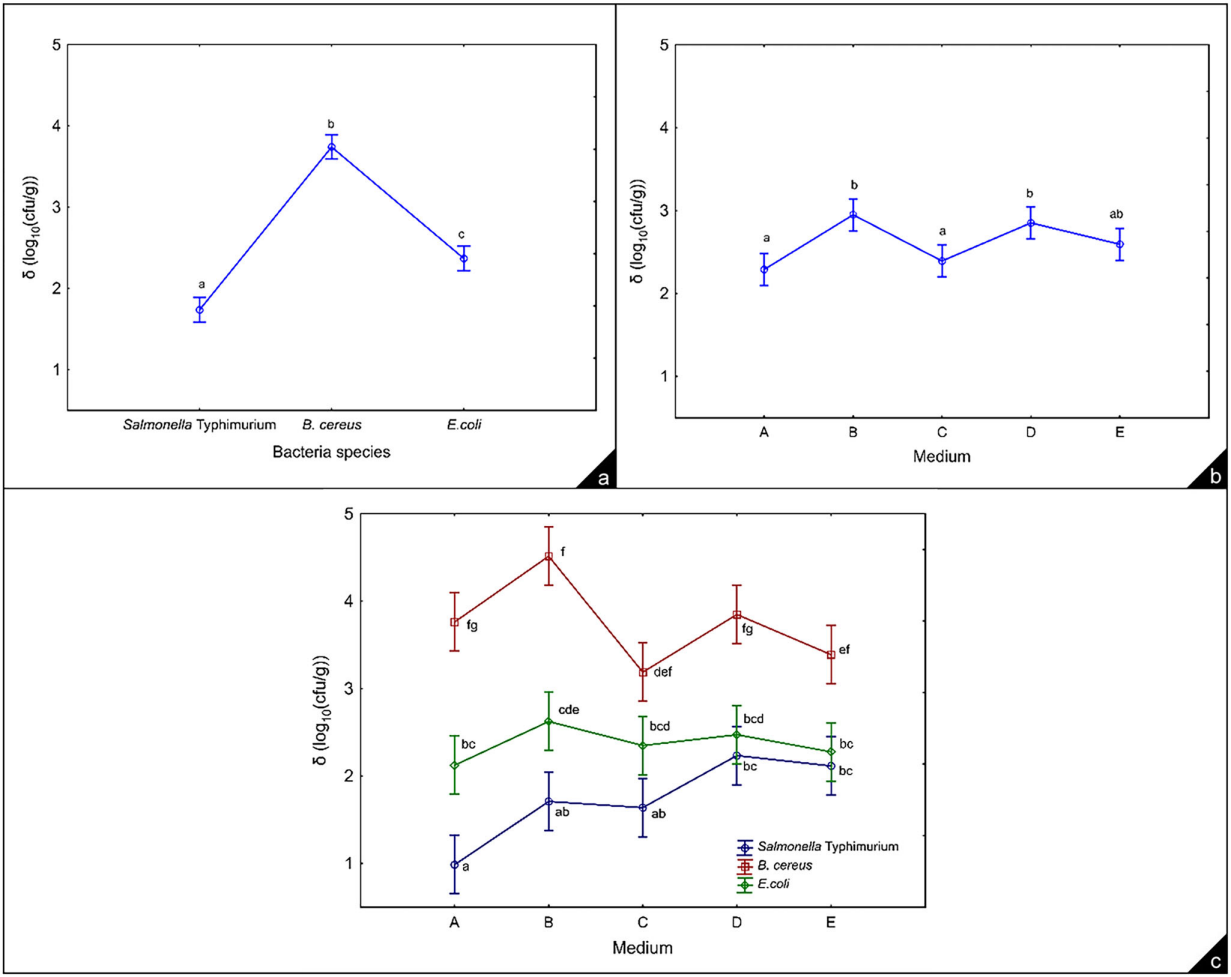
The effect of (a) bacterial species, (b) brand of cocoa‐flavored PBMAs, and (c) the interaction between bacterial species and brand of cocoa‐flavored PBMAs on the mean increase in bacterial load during 12 h of incubation at 25°C. Vertical bars indicate 0.95 confidence intervals. Distinct letter labels denote statistically significant differences (*p* < 0.05) in the increase of bacterial levels across bacterial species or PBMA type.

An ANOVA of the effects related to the brand of substrate and bacterial species showed that the increase in bacterial counts during 12 h (*δ*) of cultivation at 25°C was influenced by both factors and their interactions (Table 1). However, the type of bacterial species had a greater impact on the variability of this parameter than the brand of cocoa‐flavored plant‐based milk. Post hoc analysis using Tukey's test revealed that in the case of type of bacteria each strain forming a separate homogeneous group (Figure 2a). In turn, in the case of the medium factor, the analysis identified three homogeneous groups with respect to the increase in bacterial load (Figure 2b). In contrast, for the interaction between bacterial species and medium, as many as seven homogeneous groups were distinguished (Figure 2c), indicating a substantial differentiation in bacterial growth capacity depending on the specific combination of species and PBMA matrix.

**TABLE 1:** Results of analysis of variance (ANOVA) statistical analysis of the effect of cocoa‐flavored plant‐based milk alternatives (PBMAs), microbial species, and their interaction on the increase in bacterial load after 12 h of incubation.

Effect	df	SS	MS	*F*	*p*
Brand of cocoa‐flavored PBMAs (PBMAs)	4	1.94	0.48	9.93	<0.001
Bacterial species (BS)	2	20.97	10.49	214.35	<0.001
PBMAs × BS	8	2.36	0.29	6.02	0.001

*Note*: SS: the sum of the squares of the deviations resulting from the effects of sample and microbial species. df: the number of degrees of freedom. MS: the mean sum of the squares of the deviations resulting from the effects of sample and microbial species. *F* is the value from analysis of variance. Statistical significance level (*p* < 0.05).

The examination of the results presented in Figure 2c reveals that each of tested strain exhibits individual nutritional preferences, and the type of medium significantly affects its growth. *Salmonella* Typhimurium exhibited the lowest growth on medium A (0.99 log_10_ cfu/mL) and the highest on media D (2.23 log_10_ cfu/mL) and E (2.12 log_10_ cfu/mL), indicating that the nutrient composition of media D and E most effectively supported the growth of this strain. Intermediate values were observed on media B (1.71 log_10_ cfu/mL) and C (1.64 log_10_ cfu/mL). *E. coli* showed the highest growth on media B (2.62 log_10_ cfu/mL), D (2.47 log_10_ cfu/mL), and C (2.35 log_10_ cfu/mL), whereas the lowest growth occurred on medium A (2.12 log_10_ cfu/mL). Among all tested strains, *B. cereus* exhibited the highest overall growth across all substrates, reaching its highest values on media B (4.51 log_10_ cfu/mL) and D (3.85 log_10_ cfu/mL), and the lowest on medium C (3.19 log_10_ cfu/mL). Media A (3.76 log_10_ cfu/mL) and E (3.39 log_10_ cfu/mL) showed intermediate growth. The greater differences in *B. cereus* populations across media suggest that this strain is more sensitive to variations in medium composition than the other tested strains. The results of the analysis indicate that these substrates are susceptible to bacterial contamination and therefore require appropriate protective actions.

**FIGURE 3:**
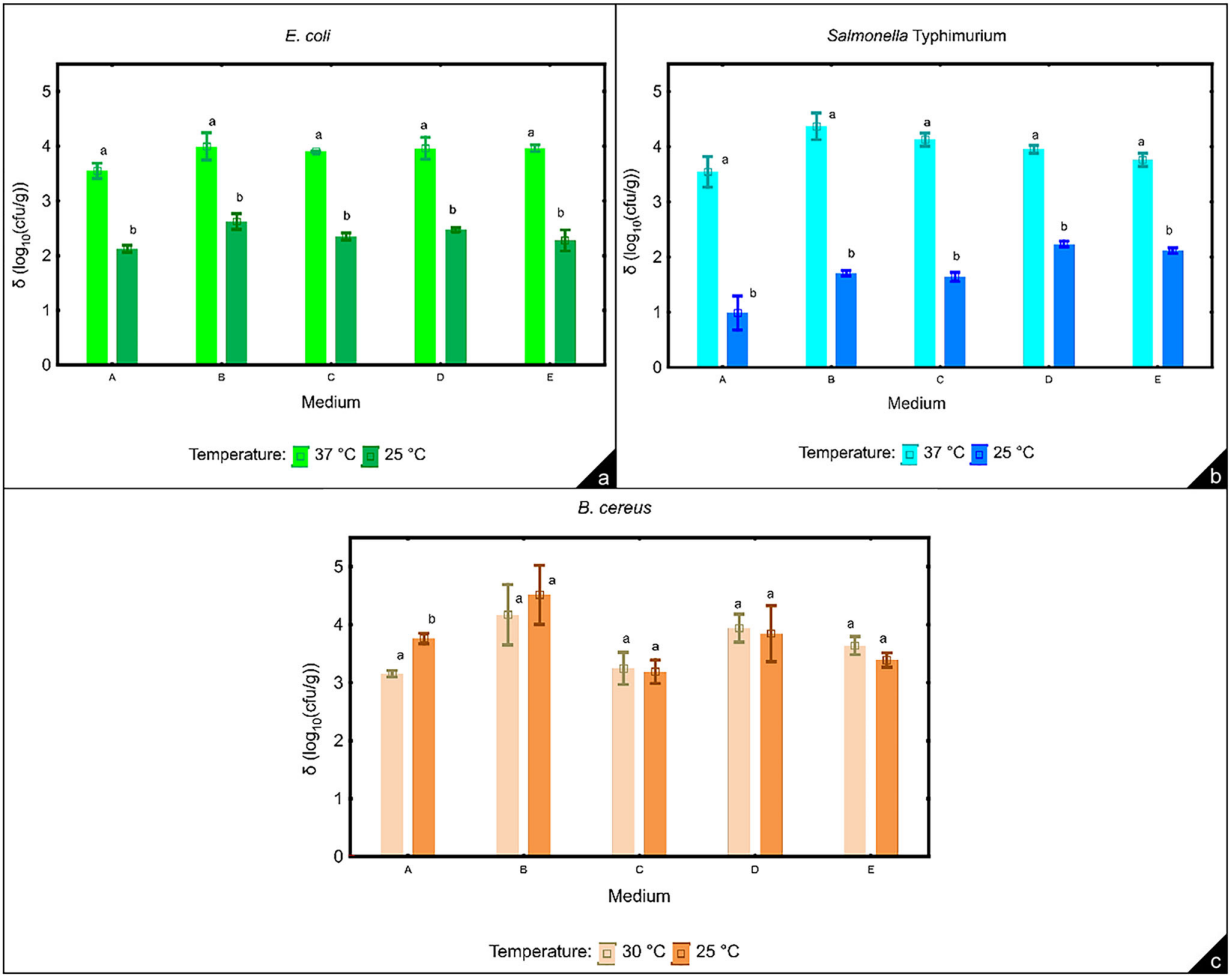
Bacterial load increase during 12‐h incubation at 25°C and 37°C (*E*. *coli* [a] and *S* *almonella* Typhimurium [b]) and at 25°C and 30°C (*B*. *cereus* [c]) on different media based on cocoa‐flavored PBMAs. Distinct letter labels within each substrate denote statistically significant differences (*p* < 0.05) in the increase of bacterial levels across the applied temperatures.

Further examination of the results showed that consistent with the known optimal growth temperatures of *S* *almonella* Typhimurium and *E. coli*, incubation at 37°C clearly promoted the growth of both mentioned types of bacteria (Figure 3). Statistical analysis revealed a significantly higher increase in the population of *S* *almonella* Typhimurium and *E. coli* at 37°C compared to 25°C on all tested cocoa‐flavored PBMAs (*p* < 0.05). Nevertheless, it is worth emphasizing that in complex food matrices such as PBMAs, microbial growth patterns may deviate due to the specific effects of matrix components and their interactions with the tested strains. In the case of *B. cereus* incubated at 30°C, the increase in its count on cocoa‐flavored PBMAs C, D, and E was slightly higher than at 25°C; however, the differences were not statistically significant (*p* > 0.05). On Samples A and B, the increases in the population of *B. cereus* were more pronounced, with higher levels observed at 25°C, although these differences were statistically significant (*p* < 0.05) only on Sample A. Together, these data demonstrate that the applied media are vulnerable to microbial growth, even at room temperature, at which these products are typically stored before being opened.

**FIGURE 4:**
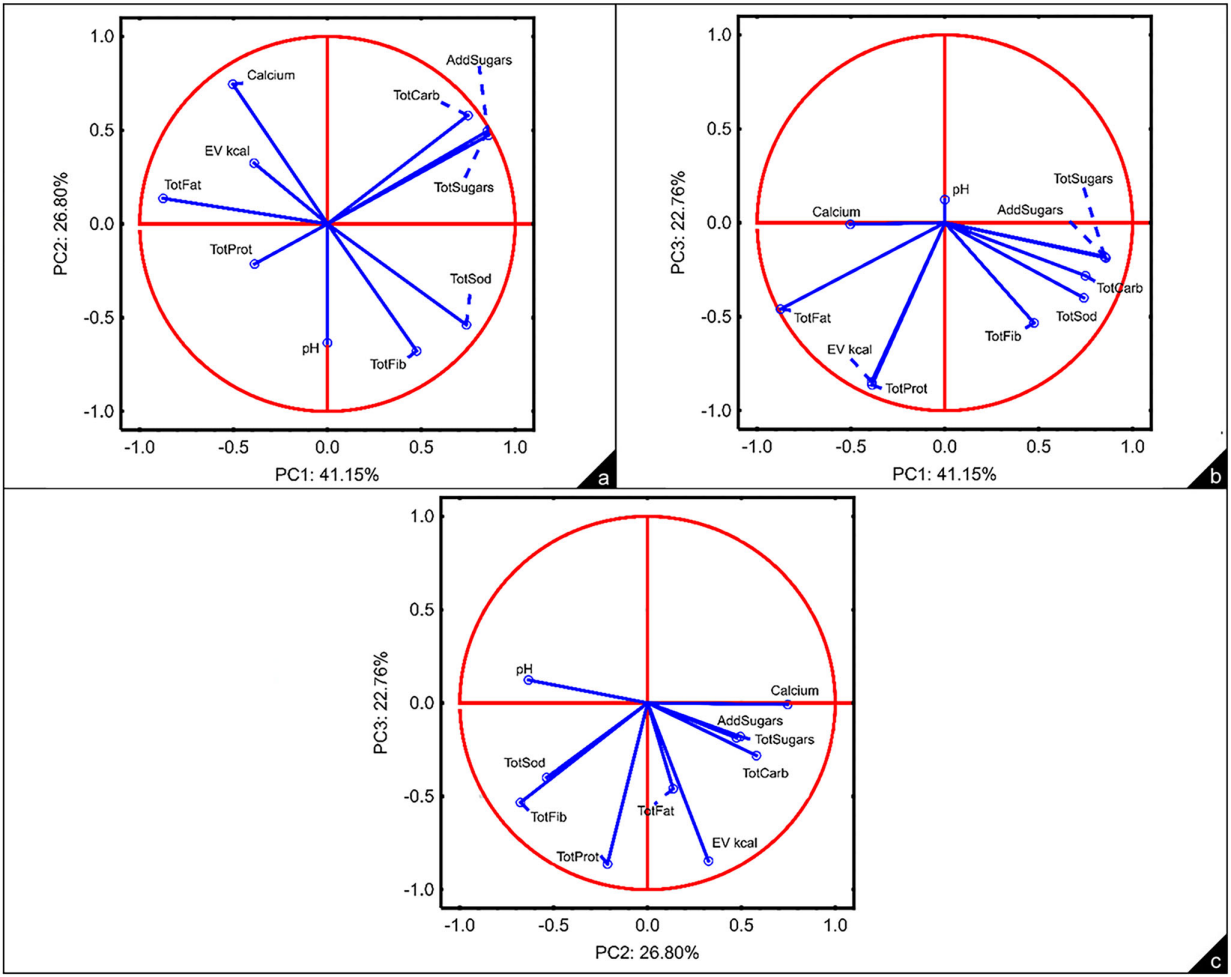
Factor maps of variables presenting the position of loading vectors for the analyzed variables describing the tested samples in the PCA component space: (a) PC1 vs PC2, (b) PC1 vs PC3, (c) PC2 vs PC3.

To better understand the effect of tested substrate composition on the growth of the examined bacterial strains, the differences among the PBMAs were further explored. Due to the complexity of the formulations and the potential relationships between variables describing their physicochemical properties (e.g., TotFat, TotProt, TotFib, TotCarb, TotSugars, AddSugars, Calcium, TotSod, EV kcal, and pH), PCA was used. This approach enabled the reduction of the dataset dimensionality while preserving the main sources of variation in the PBMAs and to identify the main components responsible for differentiating the tested substrates. The analysis revealed that the first three principal components explained a substantial proportion of the total variance, as confirmed by the scree plot and the cumulative variance criterion. These three principal components (PC1, PC2, and PC3) explained 90.7% of the total variance (Figure 4), indicating that they captured the essential variability of the original dataset and provided a reliable basis for its interpretation, reflecting the overall compositional profiles of the tested growth substrates and highlighting their similarities and differences.

The corresponding biplots provided further insights into the contribution of individual variables (Figure 4). PC1 was positively associated with total carbohydrates, added sugars, total sugar, and total sodium, while exhibiting negative loadings for total fat. This suggests that PBMAs scoring positively on PC1 were carbohydrate‐ and sodium‐rich but relatively low in fat. PC2 was defined by negative loadings for total sodium, total fiber, and pH, and positive loadings for calcium, with additional moderate contributions from carbohydrate‐related variables. Thus, PBMA samples with high PC2 values were distinguished by elevated calcium content and lower sodium, fiber, and acidity. PC3 was dominated by negative loadings for total protein, energy value, and total fiber, with weaker negative contributions from total fat and sodium. Substrates scoring negative values on PC3 were therefore characterized by higher protein and caloric content, whereas those with positive scores reflected a composition lower in these components.

**FIGURE 5:**
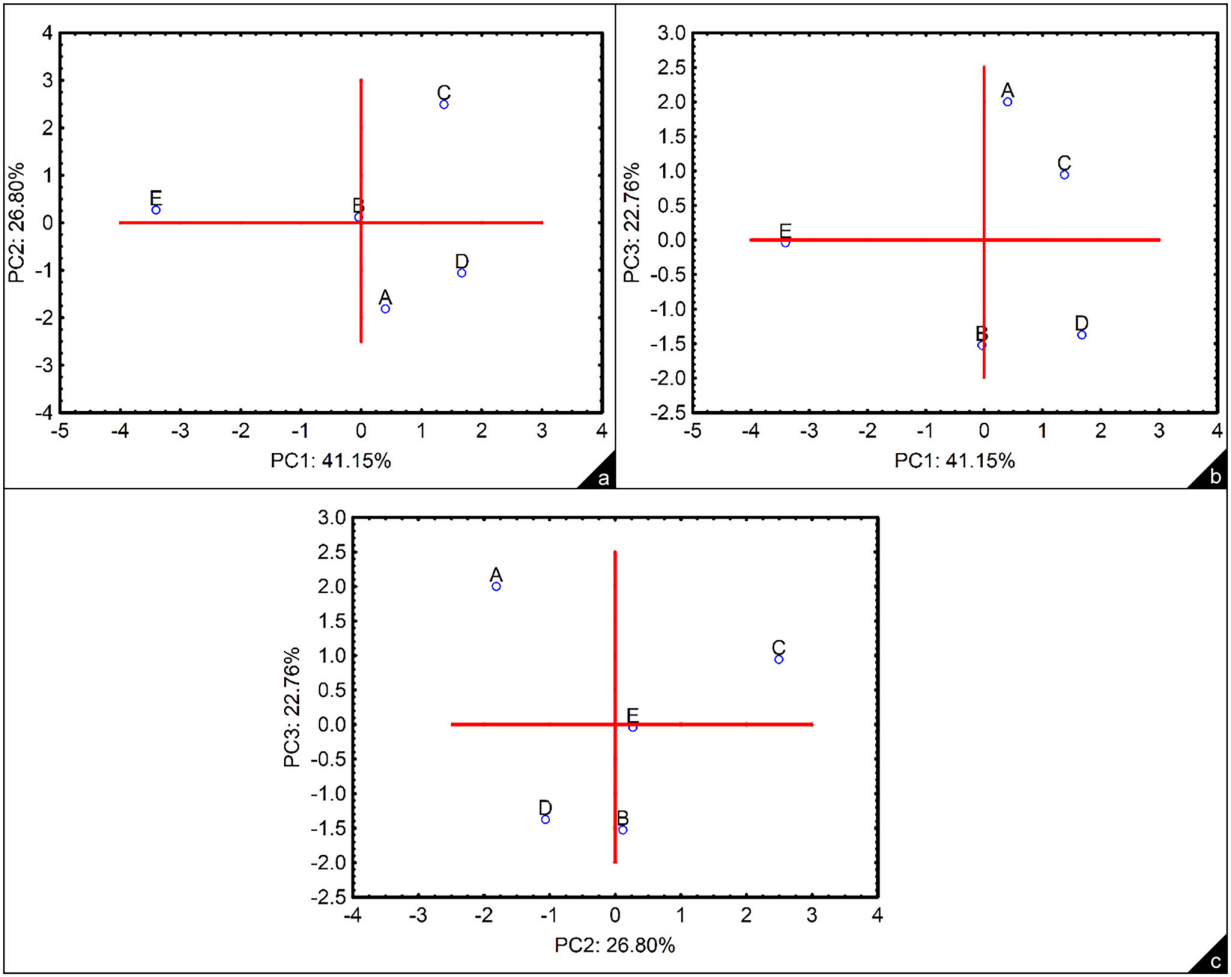
Factor coordinates of cases (substrates A, B, C, D, E) obtained from PCA analysis presented in the (a) PC1 vs. PC2, (b) PC1 vs. PC3, and (c) PC2 vs. PC3 coordinate systems.

The score plots revealed clear separation between the substrate samples (Figure 5), confirming distinct compositional differences, whereas the corresponding biplots highlighted which variables contributed most strongly to the observed separation, thus providing insights into the key factors responsible for the differentiation of the studied cocoa‐flavored PBMAs. Substrate A, located predominantly at negative PC2 and positive PC3 values, was associated with higher sodium, fiber, and pH. Substrate B, positioned mainly at negative PC3 values, was characterized by higher levels of protein, fiber, and energy, reflecting its nutrient‐dense composition. Substrate C, scoring positively across all three components, exhibited elevated concentrations of carbohydrates, sugars, sodium, and calcium, while simultaneously being relatively low in fat. Substrate D, described by positive PC1 and negative PC3 scores, combined a carbohydrate and sodium‐rich profile with higher protein, fiber, and energy content. In contrast, substrate E, distinguished by negative PC1 scores, was marked by lower levels of carbohydrates and sugars and relatively higher fat content.

The distribution pattern revealed by PCA effectively separated the samples according to their nutritional composition, highlighting the distinct roles of carbohydrate, protein‐, and mineral‐related traits in driving their differentiation. Taken together, these results demonstrate that PCA not only reduced data complexity but also highlighted the variables most responsible for distinguishing between the samples. Carbohydrate‐ and sodium‐related traits (PC1), mineral composition and acidity (PC2), and protein–energy balance (PC3) emerged as the primary axes of variation. This dimensional reduction approach allowed for a clear characterization of the substrates and provided a framework for understanding how specific nutritional factors drive their compositional differentiation. Cocoa‐derived components, including sugars, lipids, and polyphenols, may contribute to the observed growth patterns by providing nutritional support, whereas polyphenols may exert modulatory effects on microbial growth depending on their concentration and interactions within the matrix. The observed differences in bacterial growth across PBMAs can be explained by variations in substrate composition, as highlighted by the PCA results. Substrates associated with PC3, characterized by higher protein content, energy value, and fiber, are likely to promote bacterial growth due to increased nutrient availability, which supports microbial metabolism and proliferation. In contrast, PC2‐related factors, including sodium concentration and pH, may influence growth through osmotic stress and environmental conditions affecting enzyme activity and cellular homeostasis. Substrates associated with PC1, which are rich in carbohydrates and sodium but relatively low in fat, may also support growth by providing readily available energy sources; however, their contribution appears to be less pronounced compared to nutrient‐dense profiles represented by PC3.

To investigate the combined effects of environmental and substrate‐related factors on bacterial growth over time, multiple regression models were constructed for each examined strain. The regression coefficients (*β*_0_, *β*_1_,…,*β*_5_) of models determined for the analyzed strains are presented in Table 2. The formulated models describe the population increase (*ΔLog* *N*) of the three bacterial strains as a function of time, temperature, and sample composition represented by the three principal components (*PC1*, *PC2*, and *PC3*).

**TABLE 2:** Regression coefficients (*β*_0_ *–β*_5_) of models predicting bacterial population increase (*ΔLog* *N*) as a function of time (τ), temperature (T), and substrate composition (*PC1–PC3*) for three strains.

General structure of the model: $\Delta \log N=\beta_{0}+\beta_{1}\cdot \;\tau +\beta_{2}\cdot T+\beta_{3}\cdot \mathrm{PC}1+\beta_{4}\cdot \mathrm{PC}2+\beta_{5}\cdot \mathrm{PC}3$									
	*B*. *cereus*			*E*. *coli*			*Salmonella* Typhimurium		
Effect	Coefficient value	SE	*p*	Coefficient value	SE	*p*	Coefficient value	SE	*p*
*β*_0_	0.304	0.212	0.152	−1.183	0.139	0.000^*^	−2.890	0.329	<0.001^*^
*β*_1_	0.089	0.007	<0.001^*^	0.061	0.006	<0.001^*^	0.069	0.011	<0.001^*^
*β*_2_	−0.003	0.007	0.679	0.050	0.004	<0.001^*^	0.094	0.009	<0.001^*^
*β*_3_	−0.003	0.010	0.766	−0.017	0.010	0.081	−0.006	0.016	0.734
*β*_4_	−0.038	0.014	0.007^*^	0.001	0.013	0.920	0.030	0.022	0.168
*β*_5_	−0.074	0.014	0.000^*^	−0.028	0.014	0.038^*^	−0.051	0.024	0.030^*^

Abbreviation: SE standard error.

^*^Statistical significance level (*p* < 0.05).

**FIGURE 6:**
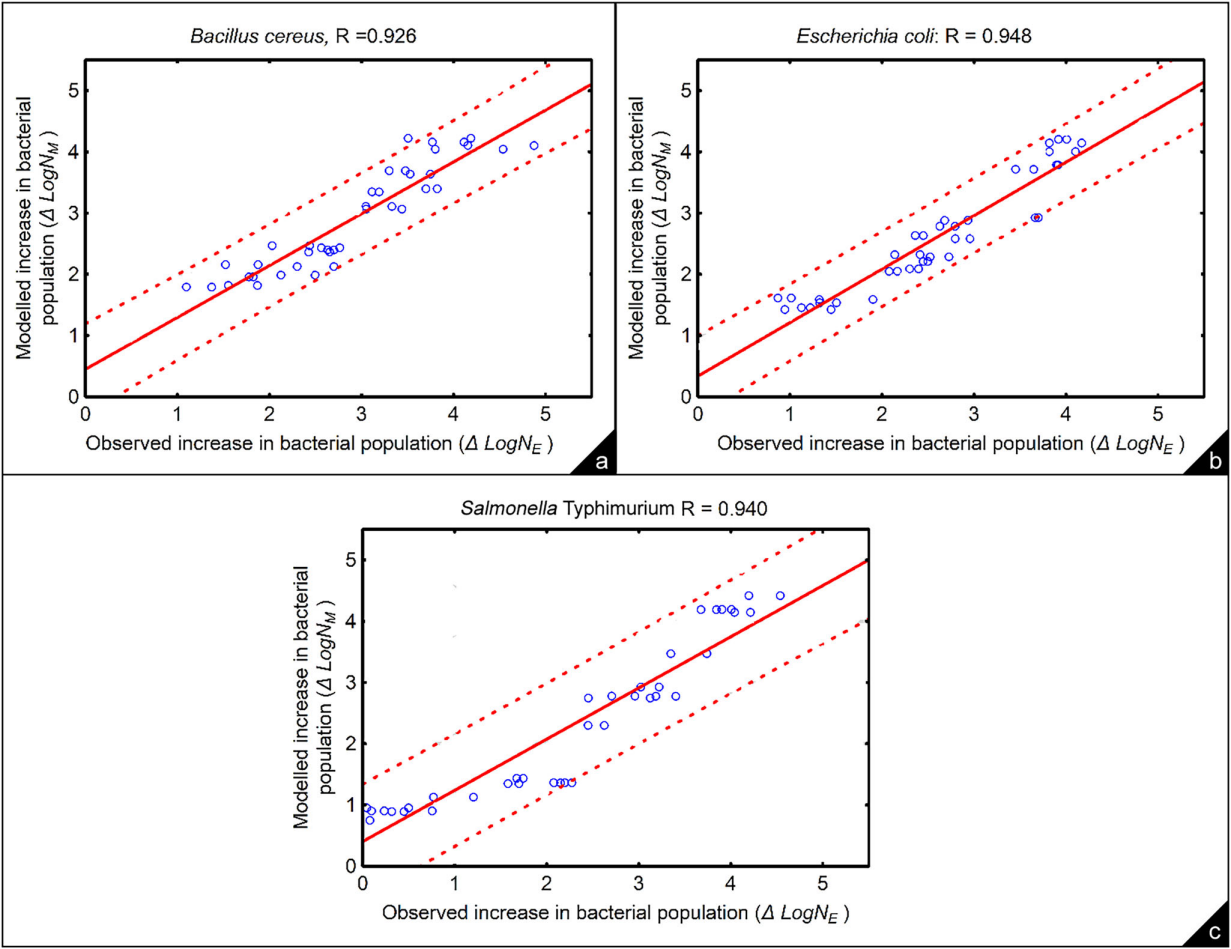
Comparison between the model predicted (*ΔLog* *N*_*M*_) and experimentally observed (*ΔLog* *N*_*E*_) increase in population size for three bacterial species: (a) *B. cereus*, (b) *E. coli* and (c) *Samonella* Typhimurium. The dashed line indicates the prediction interval.

The statistical evaluation of regression coefficients of obtained models demonstrated that factors included in their structures: time, temperature, and PBMA composition significantly influenced bacterial growth intensity (*p* < 0.05). Among the principal components, PC3 (associated mainly with Substrate B, and to a slightly lesser extent with Substrates A, C, and D) emerged as the strongest predictor of population increase across all tested bacterial strains, underscoring the important role of protein content, fiber, fat, sodium, and energy balance in shaping bacterial growth potential. In contrast, PC2 (associated with Samples A and C), which reflected the influence of sodium, fiber, and pH‐related properties, showed a strain‐specific role, exerting a strong influence on *B. cereus*, while contributing modestly to *Salmonella* Typhimurium and *E. coli*. PC1 (associated mainly with Sample E, but also with Samples C and D) plays supportive role in maintaining overall model stability (*p* > 0.05), as its exclusion from the models significantly reduced predictive accuracy of the model. A similar effect was observed for PC2 in the case of *S* *almonella* Typhimurium and *E. coli* and for temperature within the tested range for *B. cereus*. It is worth emphasizing that in predictive modeling, variable selection should not rely solely on statistical significance. Hence, in the process of model construction, predictors that appear statistically insignificant but whose presence enhances model performance should not be removed automatically, as their retention can improve the reliability of confidence intervals by preserving nominal coverage probability and stabilizing the overall structure of the model (Harrell 2016). Figure 6 shows the comparison between the bacterial population increase predicted by the model (*ΔLog* *N*_*M*_) and the experimentally observed increase (*ΔLog* *N*_*E*_).

Model evaluation was carried out by comparing observed and predicted population levels across the experimental datasets from which it was derived. Correlation plots between observed and predicted values demonstrated a strong agreement between the model outputs and the empirical data (Table 3). The correlation coefficients (*R* = 0.92–0.94) reflected a strong positive linear relationship, indicating that the models successfully reproduced most of the variability present in the actual observations. The explanatory power of the regression models, expressed by the coefficient of determination (*R* ^2^), was also high, ranging from 0.857 to 0.898 depending on the bacterial strain. These results suggest that the models accounted for most of the variability in bacterial population growth. Low RMSE and MAE values also indicate that the model accurately reproduced the observed data.

**TABLE 3:** Statistical criteria used to evaluate the prediction accuracy of models describing the increase in bacterial population (*ΔLog* *N*) of three bacterial strains as a function of time, temperature, and sample composition represented by the three principal components (*PC1*, *PC2*, and *PC3*).

Bacterial strain	Coefficient of determination	Root mean square error	Mean absolute error
	*R* ^2^	RMSE	MAE
*B*.T *cereus*	0.857	0.346	0.281
*E*. *coli*	0.898	0.316	0.251
*S* *almonella* Typhimurium	0.884	0.494	0.411

Abbreviations: MAE, mean absolute error; RMSE, root mean square error.

**FIGURE 7:**
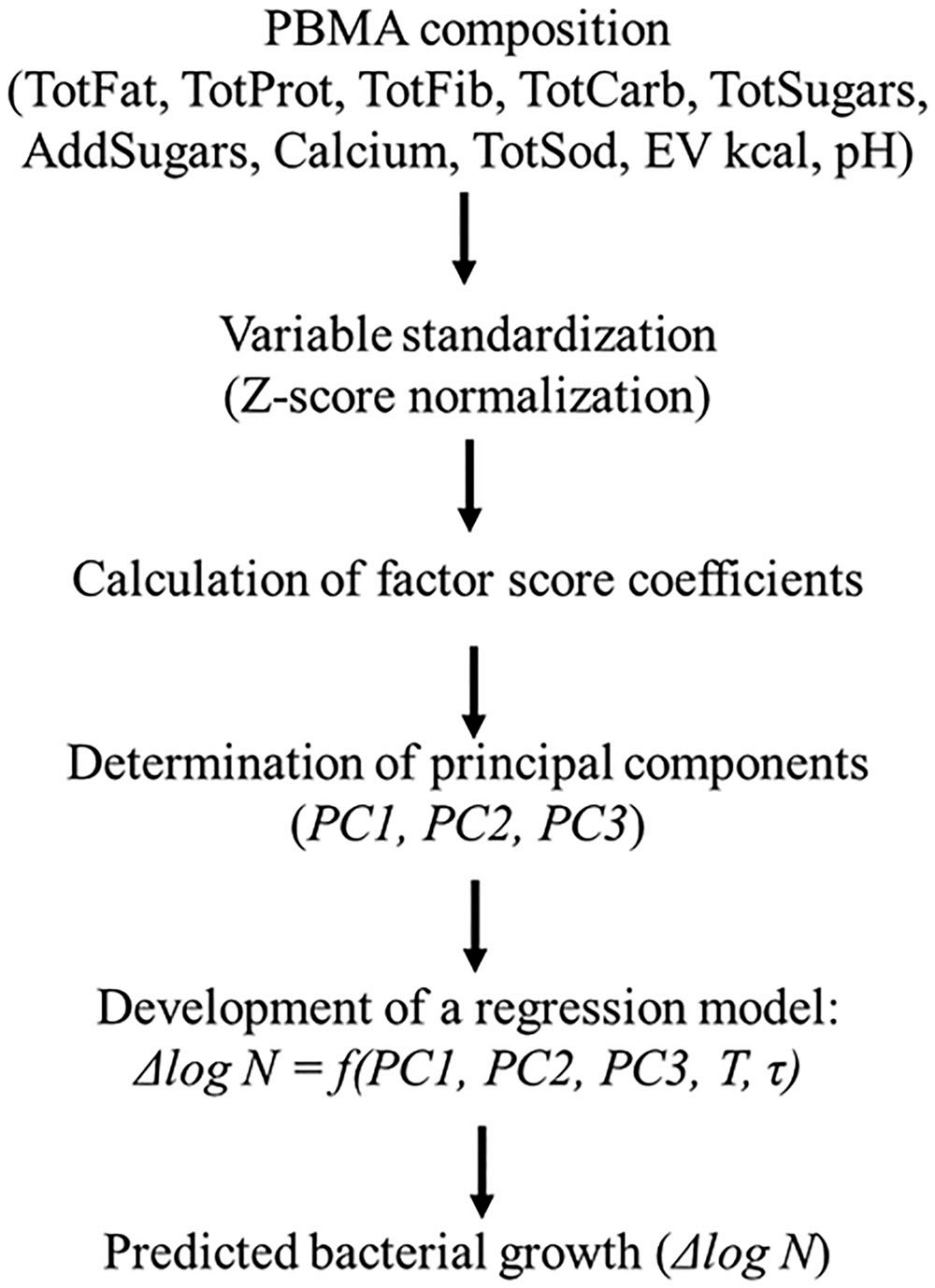
Framework for mapping PBMA composition into PCA space and predicting bacterial growth using the developed regression model. PBMA, plant‐based milk alternative.

Overall, the regression models not only provided accurate estimates of bacterial growth under different experimental conditions but also highlighted the relative contribution of PBMAs characteristics, expressed through their loadings on the principal components, to strain‐specific growth patterns. The developed model can be readily applied to new PBMA formulations. As it is presented in Figure 7, to ensure consistency, the raw compositional variables must first be standardized according to Equation (2) using the mean and SD derived from the training dataset (Table 4). Then, based on the factor score coefficients computed for the training dataset (Table 4), the standardized variables can be projected into the PCA space as follows:

(4) $$PC_{k}=\sum_{i=1}^{n}a_{ki}\cdot z_{i}$$

where *a*_k_ *_i_* represents the regression‐based factor score coefficients, and *z_i_* are standardized compositional variables. The resulting principal components (*PC1*–*PC3*) constitute then inputs to the regression model to predict bacterial growth under specified environmental conditions. This allows product developers to screen formulation options and prioritize combinations that are likely to be safer, without needing to test every possible ingredient ratio experimentally.

**TABLE 4:** Means and standard deviations (SDs) together with factor score coefficients derived from the training dataset used for principal component analysis (PCA)‐based transformation of plant‐based milk alternative (PBMA) compositional variables.

Variable (ingredient)	Factor score coefficients for:			Mean	SD
	PC1	PC2	PC3		
EV kcal (200 mL)	−0.095060	0.121018	−0.371760	96.800	20.6688
TotCarb	0.181913	0.216099	−0.123740	11.3000	3.1241
TotSugars	0.206751	0.184334	−0.078910	8.3600	5.4656
AddSugars	0.208167	0.176358	−0.081910	7.6600	5.4331
TotProt	−0.094400	−0.079860	−0.378850	2.5600	1.0691
TotFat	−0.212740	0.050730	−0.201790	4.0600	2.2777
TotSod	0.179804	−0.200860	−0.175590	0.0684	0.0508
Calcium	−0.122590	0.278375	−0.003410	0.3514	0.0683
TotFib	0.115328	−0.252780	−0.234110	2.5180	1.3189
pH	−0.000070	−0.236780	0.054044	0.0148	0.0086

In this context, the model can be regarded as a data‐driven tool for reducing microbial risk in PBMAs based on patterns identified from prior experiments. The study findings provide a valuable framework for understanding how environmental and nutritional variables jointly regulate bacterial growth intensity and may support the development of predictive tools for microbial risk assessment in applied food and feed systems. This methodology can serve as a guide for predicting the bacterial growth; however, when the analyzed beverage substrate differs substantially from the original PBMAs, the predictions should be experimentally verified to ensure their accuracy before practical application.

## 4 Conclusion

This study demonstrated that the composition of individual PBMAs strongly influences pathogen behavior. Among the compositional factors, protein, fiber, fat, sodium, and energy content emerged as key nutritional drivers promoting bacterial growth. Temperature was also identified as a critical determinant, with 37°C significantly enhancing the growth of *E. coli* and *S* *almonella* Typhimurium, while affecting *B. cereus* in a substrate‐specific manner. PCA reveals three main dimensions of substrate differentiation reflecting: nutrient density (protein, energy, and fiber)—PC3, mineral content and pH—PC2, and the balance between carbohydrates and fat—PC1. Each substrate exhibits a distinct profile across these dimensions, allowing a clear separation of their contributions to the observed effects. Regression models based on PCA scores accurately captured growth patterns, highlighting the combined effects of environmental and nutritional factors. Overall, these findings underscore the importance of simultaneous considering formulation characteristics and storage conditions to ensure the microbial safety of cocoa‐flavored PBMAs and provide a framework for developing predictive tools to support microbial risk assessment in plant‐based beverages.

## Author Contributions

**Clara Mariana Gonçalves Lima**: conceptualization, investigation, writing – original draft, writing – review and editing, visualization, validation, methodology, formal analysis, data curation. **Jaqueline Sousa Correia**: investigation, writing – original draft, methodology, writing – review and editing, formal analysis, visualization. **Dionísio Pedro Amorim‐Neto**: investigation, writing – original draft, writing – review and editing, visualization, formal analysis. **Jolanta Wawrzyniak**: conceptualization, investigation, writing – original draft, writing – review and editing, visualization, validation, methodology, software, formal analysis, data curation, resources. **Anderson S. Sant'Ana**: conceptualization, investigation, funding acquisition, writing – original draft, writing – review and editing, visualization, validation, methodology, project administration, formal analysis, supervision, resources.

## Conflicts of Interest

The authors declare no conflicts of interest.

## References

[jfds71189-bib-0002] Abebe, E. , G. Gugsa , and M. Ahmed . 2020. “Review on Major Food‐Borne Zoonotic Bacterial Pathogens.” Journal of Tropical Medicine 2020, no. 1: 4674235. 10.1155/2020/4674235. 32684938 PMC7341400

[jfds71189-bib-0024] AFSSA (Agence Française de Sécurité Sanitaire des Aliments) . 2004. Compte rendu des essais ISSO sur l’incertitude de mesure 2003/2004. France: AFSSA.

[jfds71189-bib-0003] Amorim‐Neto, D. P. , C. M. G. Lima , S. D. N. M. Ramos , et al. 2025. “The Culturable Microbiological Landscape of Guarana‐Based Products and Wastes: Insights From the Amazon State (Brazil) Production Chain.” Applied Food Research 5, no. 2: 101162. 10.1016/j.afres.2025.101162.

[jfds71189-bib-0001] ANVISA (Agência Nacional de Vigilância Sanitária) . 2005. Padronização dos Testes de Sensibilidade a Antimicrobianos por Disco‐difusão: Norma aprovada. 8th ed. ANVISA.

[jfds71189-bib-0004] Butovskaya, E. , E. Caprai , M. Peloso , et al. 2025. “Plant‐Based Milk Alternatives: Assessing the Occurrence of Chemical and Microbiological Contaminants in Soy, Oat, Rice and Almond Beverages From Italian Market.” Food Control 169: 111005. 10.1016/j.foodcont.2024.111005.

[jfds71189-bib-0005] Champidou, C. , M. Ellouze , M. Campagnoli , O. Robin , N. Haddad , and J.‐M. Membré . 2024. “Unveiling the Matrix Effect on *Bacillus licheniformis* and *Bacillus subtilis* Spores Heat Inactivation Between Plant‐Based Milk Alternatives, Bovine Milk and Culture Medium.” International Journal of Food Microbiology 422: 110807. 10.1016/j.ijfoodmicro.2024.110807. 38970999

[jfds71189-bib-0006] Correia, J. S. , D. P. Amorim‐Neto , E. Lang , et al. 2026. “Microbiological Quality of Plant‐Based Cheese Analogues and Pathogen Behavior in Cashew Nut‐Based Varieties in Cashew Nut‐Based Varieties.” International Journal of Food Microbiology 451: 111670. 10.1016/j.ijfoodmicro.2026.111670. 41666777

[jfds71189-bib-0025] EURL Lm (European Union Reference Laboratory for Listeria monocytogenes) . 2008. Technical guidance document on shelf‐life studies for Listeria monocytogenes in ready‐to‐eat foods. SANCO/1628/2008 ver. 9.3 (26112008). Working document, November 2008. Europe: EURL Lm.

[jfds71189-bib-0007] Gleissle, A. , H. Schmidt , and J. Hinrichs . 2025. “Prevalence of Spore‐Forming Bacteria in Plant‐Based Raw Materials Used for Plant‐Based Milk Alternatives.” International Journal of Food Microbiology 439: 111255. 10.1016/j.ijfoodmicro.2025.111255. 40378488

[jfds71189-bib-0008] Gonçalves Lima, C. M. , D. P. Amorim‐Neto , B. G. de Castro , et al. 2026. “Microbiological Characterization of Baru Nuts From the Cerrado Biome, Brazil.” Journal of Food Science 91, no. 4: e71043. 10.1111/1750-3841.71043. 41960607 PMC13067225

[jfds71189-bib-0009] Grau‐Fuentes, E. , D. Rodrigo , R. Garzón , and C. M. Rosell . 2023. “Understanding the Marketed Plant‐Based Beverages: From Ingredients Technological Function to Their Nutritional Value.” Journal of Functional Foods 106: 105609. 10.1016/j.jff.2023.105609.

[jfds71189-bib-0027] Harrell Jr., F. E. 2016. Regression modeling strategies. Springer Series in Statistics. Cham, Switzerland: Springer International Publishing.

[jfds71189-bib-0026] Instituto Adolfo Lutz . 2005. Métodos físico‐químicos para análise de alimentos: normas analíticas do Instituto Adolfo Lutz. 4th ed. Brasília, Brazil: Agência Nacional de Vigilância Sanitária.

[jfds71189-bib-0010] Irondi, E. A. , H. T. Aina , Y. T. Imam , et al. 2025. “Plant‐Based Milk Substitutes: Sources, Production, and Nutritional, Nutraceutical and Sensory Qualities.” Frontiers in Food Science and Technology 5: 1593870. 10.3389/frfst.2025.1593870.

[jfds71189-bib-0011] Jeske, S. , E. Zannini , and E. K. Arendt . 2018. “Past, Present and Future: The Strength of Plant‐Based Dairy Substitutes Based on Gluten‐Free Raw Materials.” Food Research International 110: 42–51. 10.1016/j.foodres.2017.03.045. 30029705

[jfds71189-bib-0012] Kain, T. , M. Albahri , M. Plötz , and N. Jessberger . 2024. “Growth, Persistence and Toxin Production of Pathogenic Bacteria in Plant‐Based Drinking Milk Alternatives.” Journal of Food Science 89, no. 9: 5799–5811. 10.1111/1750-3841.17309. 39169550

[jfds71189-bib-0013] Kasapidou, E. , Z. Basdagianni , G. Papatzimos , et al. 2023. “Chemical Composition, Antioxidant Profile and Physicochemical Properties of Commercial Non‐Cocoa‐ and Cocoa‐Flavoured Plant‐Based Milk Alternatives.” European Food Research and Technology 249, no. 12: 3011–3026. 10.1007/s00217-023-04345-3.

[jfds71189-bib-0014] Kyrylenko, A. , R. T. Eijlander , G. Alliney , E. L.‐V. de Bos , and M. H. J. Wells‐Bennik . 2023. “Levels and Types of Microbial Contaminants in Different Plant‐Based Ingredients Used in Dairy Alternatives.” International Journal of Food Microbiology 407: 110392. 10.1016/j.ijfoodmicro.2023.110392. 37729802

[jfds71189-bib-0015] Lee, P. Y. , S. Y. Leong , and I. Oey . 2024. “The Role of Protein Blends in Plant‐Based Milk Alternative: A Review Through the Consumer Lens.” Trends in Food Science & Technology 143: 104268. 10.1016/j.tifs.2023.104268.

[jfds71189-bib-0016] Lima, C. M. G. , D. P. Amorim‐Neto , N. Hennig Neuenfeldt , et al. 2025. “Microbiological Contamination and Mycotoxin Detection in Guarana Integument: Addressing Safety Concerns in Guarana Agro‐Industrial Co‐Products.” Food Science and Technology 45: e500. 10.5327/fst.500.

[jfds71189-bib-0017] McClements, D. J. , E. Newman , and I. F. McClements . 2019. “Plant‐Based Milks: A Review of the Science Underpinning Their Design, Fabrication, and Performance.” Comprehensive Reviews in Food Science and Food Safety 18, no. 6: 2047–2067. 10.1111/1541-4337.12505. 33336952

[jfds71189-bib-0018] Misiou, O. , K. Koutsoumanis , and J.‐M. Membré . 2023. “Quantitative Microbial Spoilage Risk Assessment of Plant‐Based Milk Alternatives by *Geobacillus stearothermophilus* in Europe.” Food Research International 166: 112638. 10.1016/j.foodres.2023.112638. 36914335

[jfds71189-bib-0019] Mukuna, W. , A. I. Mafiz , B. Pokharel , A. Tobenna , and A. Kilonzo‐Nthenge . 2021. “Antibiotic Resistant Enterobacteriaceae in Milk Alternatives.” Foods 10, no. 12: 3070. 10.3390/foods10123070. 34945621 PMC8702211

[jfds71189-bib-0020] Paul, A. A. , S. Kumar , V. Kumar , and R. Sharma . 2020. “Milk Analog: Plant Based Alternatives to Conventional Milk, Production, Potential and Health Concerns.” Critical Reviews in Food Science and Nutrition 60, no. 18: 3005–3023. 10.1080/10408398.2019.1674243. 31617734

[jfds71189-bib-0021] Precedence Research . 2025. Dairy Alternatives Market Size, Share, and Trends 2025 to 2035. Precedence Research. https://www.precedenceresearch.com/dairy‐alternatives‐market.

[jfds71189-bib-0022] Sethi, S. , S. K. Tyagi , and R. K. Anurag . 2016. “Plant‐Based Milk Alternatives an Emerging Segment of Functional Beverages: A Review.” Journal of Food Science and Technology 53, no. 9: 3408–3423. 10.1007/s13197-016-2328-3. 27777447 PMC5069255

[jfds71189-bib-0023] Vogelsang‐O'Dwyer, M. , E. Zannini , and E. K. Arendt . 2021. “Production of Pulse Protein Ingredients and Their Application in Plant‐Based Milk Alternatives.” Trends in Food Science & Technology 110: 364–374. 10.1016/j.tifs.2021.01.090.

